# Surgical reintervention requirements following GreenLight PVP: A single-center experience using three different laser device models

**DOI:** 10.1080/2090598X.2023.2222262

**Published:** 2023-06-10

**Authors:** Bora Özveren, Nejdet Karşıyakalı, Levent Türkeri

**Affiliations:** Department of Urology, School of Medicine, Acıbadem Mehmet Ali Aydınlar University, Istanbul, Turkey

**Keywords:** Greenlight, Photovaporization, Benign prostate hyperplasia, Bladder neck contracture, Urethral stricture

## Abstract

**Objective:**

To assess the incidence, risk factors, and timing of specific causes of reoperations following PVP.

**Material and Methods:**

A retrospective analysis of data on men who underwent GreenLight PVP between 2004 and 2019 in a single center and required surgical intervention for bladder neck contracture (BNC), urethral stricture (US), or persistent/recurrent prostate adenoma.

**Results:**

The overall rate of reoperations was 13.8% during a 61-month median follow-up of 377 patients. Reoperations were due to BNC, US, and adenoma in 7.7%, 5.6%, and 4.8% of cases, respectively. The median interval until reoperation for US (11 months) was significantly shorter. None of the risk factors had any relevance to US. In patients who underwent reoperation for BNC, lasing time and energy were significantly lower, and the prostate volume was smaller; however, the multivariate analysis only identified shorter lasing time as a predictor. In patients who had reoperation for persistent/recurrent adenoma, the PSA was increased, while the prostate volume was non-significantly high, and performance by less-experienced surgeons was associated with a higher rate of reoperations (*p* < 0.05). A longer lasing time predicted an increased risk of reoperation for adenoma in multivariate analysis.

**Conclusions:**

An unselective utilization of PVP may result in a relatively high rate of reoperations. The correlation of BNC with shorter lasing time may imply a higher risk after PVP of smaller prostates. A longer lasing time predicts an increased risk of reoperation due to persistent/recurrent adenoma, which may be related to higher prostate volumes and inefficient PVP by less-experienced surgeons.

## Introduction

GreenLight^TM^ Laser Photoselective vaporization of the prostate (PVP) (*American Medical System*, Minnetonka, MN/*Boston Scientific*, Marlborough, MA, USA) has been an attractive alternative to TURP, with excellent intraoperative safety and shorter hospitalization. Although PVP has an efficacy comparable to TURP, the reoperation rate is of concern, varying from 1.1% to 13.3% in contemporary series [1]. Re-treatments are usually required because of urethral stricture (US), bladder neck contracture (BNC), and persisting/recurrent adenoma, though specific causes are not elucidated in all studies [2].

Three different PVP device models have been in use since the introduction of the 80W KTP laser in 2002 [3]. Higher-powered devices were introduced in 2006 (120W-HPS) and 2013 (180W-XPS) to provide higher energy output, thus increasing the amount of vaporized tissue within a shorter period and generating improved outcomes.

At our institution, we have had experience with all three models of GreenLight^TM^ Laser devices consecutively since 2004 [4]. Although short-term safety and efficacy of PVP in the treatment of BPH were evident, the reoperation rates have remained a concern for the durability of the functional results and patient satisfaction in our experience.

The purpose of this study was to assess the surgical reinterventions required after PVP due to BNC, US, and obstructing prostatic enlargement in patients who were treated and followed up in the same department. We also investigated the time-to-event, the impact of different PVP laser device models, and specific perioperative clinical parameters on each cause of redo procedures.

## Material and methods

This is a retrospective observational analysis of prospectively collected data on consecutive men undergoing PVP with the Greenlight KTP, HPS, and XPS laser devices between 2004 and 2019 in our center. The indication for surgery was bothersome lower urinary tract symptoms associated with prostate enlargement not responding to medical therapy.

We included patients operated with Greenlight laser devices only. Prostate volume was not deemed as a selection criterion for PVP, and the choice of using PVP for surgical treatment of BPH was at the surgeon’s discretion. The presence of prostate cancer, evidence of neurogenic bladder dysfunction, history of previous urethral stricture/bladder neck stenosis, as well as those with strictures detected during urethroscopy and who had undergone multiple concomitant transurethral operations were excluded.

The study protocol was approved by the Acıbadem University and Acıbadem Healthcare Institutions Medical Research Ethics Committee (ATADEK) (2020–13/2, 25.6.2020).

All PVP procedures were performed by a total of 8 surgeons, highly competent at transurethral resection procedures but with disparate experiences in PVP. A 22.5F GreenLight continuous irrigation laser cystoscope (Richard Wolf GmbH, Knittlingen, Germany) was used in all cases. Anatomical vaporization by GreenLight laser was performed according to the surgical technique preferred by the operator. The power settings were limited by the model of the device used in each procedure. During the procedure, the power was titrated for optimum vaporization and safety, at the discretion of the operator. All patients were operated on under general or regional anesthesia and had a urinary catheter postoperatively with continuous bladder irrigation.

### Assessments

The patient’s pre-operative PSA, prostate volume (determined by transabdominal or transrectal ultrasonography), and ASA scores were recorded. Intraoperative lasing time, the amount of energy (kJ) administered by the laser source, and post-operative catheterization time were documented. The cohort was analyzed in subgroups based on the utilization of different PVP devices and operation by one of the less-experienced (<100 cases) 7 surgeons versus more-experienced (>100 cases) 1 surgeon.

Postoperatively, patients were followed up at the discretion of their physician. The details about the re-operations were documented by procedural notes in electronic health records. Our retrospective investigation consisted only of re-do surgical procedures and did not document other interventions such as urethral meatal dilatations or intermittent catheterization.

### Statistical analysis

The NCSS Software (Utah, USA) program was used for statistical analysis. Shapiro Wilk test and box plot graphs were used in the normal distribution conformity of variables as well as descriptive statistical methods (Mean, Standard deviation, median, frequency, ratio) when evaluating the study data. Student t-test in cross-group comparisons of variables with normal distribution; Mann Whitney U test was used in cross-group comparisons of variables that did not show normal distribution. Pearson Chi-Square test and Fisher–Freeman Halton test were used to compare qualitative data. Statistical significance was set at p<0.05 level in outcome analysis (Table 2).

**Table 1.** Baseline characteristics and perioperative data of 377 patients who have undergone GreenLight PVP.

		Mean+SD	Median (IQR)
Age (n=377)		63.9±8.3	64 (58–70)
Prostate Volume (ml) (n=335)		56.4±27.7	50 (35–70)
Pre-operative PSA (ng/ml) (n=347)		3.2±3.4	2.2 (1.2–3.9)
Lasing time (minutes) (n=340)		35.95±15.48	31 (10–120)
Applied energy (Joule) (n=344)		206.7±104	192 (133.5–256)
Applied Lasing Density (J/ml) (n=312)		4.0±2.2	3.6 (2.6–4.8)
Catheterization time (days) (n=377)		1.2±0.5	1 (1–1)
Time to reoperation for bladder neck contracture (months)		45.3±45	25 (7–78.5)
Time to reoperation for urethral stricture (months)		21.5±25.6	9 (4–36)
Time to reoperation for persistent/recurrent adenoma (months)		63.33±43,30	57 (26–95)
Overall Follow-up (months) (n=377)		68.2±44.2	61 (31–108)
Reoperation-free Follow-up (months)		63.29±43.31	57 (1–193)
		**N**	**%**
Surgeon experience	>100 cases	238	63.1
	<100 cases	139	36.9
ASA	1	354	93.9%
	2	22	5.8%
	3	1	0.3%
GreenLight^TM^ PVP Device	80W	72	19.1%
	120W	198	52.5%
	180W	107	28.4%
Bladder Neck Contracture		29	7.7%
Urethral Stricture		21	5.6%
Reoperation for persistent/recurrent adenoma		18	4.8%
Overall reoperation		52	13.8%

**Table 2.** Outcome analysis of factors associated with reoperations.

		Age	Prostate volume (mL)	Pre-operative PSA (ng/mL)	Lasing time (minute)	Applied energy (Joule)	Applied Lasing density (J/mL)	Catheter time (day)	Overall Follow-up (month)	Retreat ment-free survival Median (IQR) (month)	Surgeon experience		ASA Score			GreenLight^TM^ PVP Device		
											>100 cases	<100 cases	I	II	III	80W	120W	180W
All Reoperations	yes (n=52)	63.3±8.4	47(31–70) (31–70)	1.9 (1.2–3.7)	32.4±12.6	173.7±73.1	3.5±1.7	1.2±.5	92 (37–120)	36(40–120) (40–120)	35 (67.3)	17 (32.7)	49 (94.2)	3 (5.8)	0	11 (21.1)	29 (55.7)	12 (23.1)
	no (n=325)	64.0±8.30	50(36–71) (36–71)	2.2(1.2–4) (1.2–4)	36.5±15.8	211.9±107.2	4.1±2.2	1.2±0.5	59(30–96) (30–96)	59 (30–96)	203 (62.5)	122 (37.5)	305 (93.8)	19 (5.9)	1 (0.3)	61 (18.8)	169 (52.0)	95 (29.2)
	*p*-value	^†^ 0.581	^‡^.338	^‡^.907	^†^ 0.095	^†^ .003*	^†^ .098	^†^ 0.466	^‡^.032*	^‡^0.001*	^§^.501		^¶^1.000			^§^.653		
Bladder Neck Contracture	yes (n=29)	63.06±8.24	41(29–64) (29–64)	1.7 (1.0–3.2)	26.85±6.01	159.70±58.45	3.81±1.96	1.24±.63	83 (34–115)	19(7–76) (7–76)	23 (79.3)	6(2.7) (20.7)	26 (89.7)	3 (10.3)	0	4 (13.8)	17 (58.6)	8 (27.6)
	no (n=348)	63.94±8.32	50(36–73) (36–73)	2.2(1.2–4) (1.2–4)	36.7±15.8	21.7±106.1	4.04±2.19	1.22±0.52	60(31–104) (31–104)	58 (29.5–96)	215 (61.8)	133 (38.2)	328 (94.3)	19 (5.4)	1 (0.3)	68 (19.5)	181 (52.0)	99 (28.4)
	*p*-value	^†^ 0.585	^‡^.092	^‡^.351	^†^0.001*	^†^.001*	^†^.641	^†^0.844	^‡^.234	^‡^0.001*	^§^.060		^¶^0.289			^§^.707		
Urethral Stricture	yes (n=21)	66(61–68) (61–68)	49.5(33–71.5) (33–71.5)	2 (1–4.8)	29(21.5–40) (21.5–40)	153.5(120–255) (120–255)	2.9 (2.6–3.8)	1 (1–1)	66 (12–118)	11(4.5–42.0) (4.5–42.0)	14 (66.7)	7 (33.3)	19 (90.5)	2 (9.5)	0	4 (19.0)	10 (47.6)	7 (33.3)
	no (n=326)	63.5 (57.5–70)	50(35.5–71) (35.5–71)	2.2(1.2–3.9) (1.2–3.9)	31 (25–45)	192(135–256) (135–256)	3.6(2.6–4.8) (2.6–4.8)	1 (1–1)	6.5(34–106) (34–106)	58.5 (28.3–96)	224 (62.9)	132 (37.1)	335 (94.1)	20 (5.6)	1 (0.3)	68 (19.1)	188 (52.8)	100 (28.1)
	*p*-value	^‡^0.282	^‡^.757	^‡^.971	^‡^0.199	^‡^.175	^‡^.161	^‡^0.607	^‡^.874	0.001*	^§^.730		^¶^0.739			^§^.863		
Persistent/Recurrent Adenoma	yes (n=18)	62.5(56.7–68) (56.7–68)	64(49–82.7) (49–82.7)	3.4 (1.6–6.8)	45(34.3–50) (34.3–50)	199(121.3–272.3) (121.3–272.3)	2.8 (2–4)	1 (1–1)	114 (97–134)	57.5(37.5–95.2) (37.5–95.2)	7 (38.9)	11 (61.1)	17 (94.4)	11 (5.6)	0	7 (38.9)	9 (50.0)	2 (11.1)
	no (n=359)	64 (58–70)	50(35–70) (35–70)	2.2(1.2–3.8) (1.2–3.8)	30 (25–45)	192(134–256) (134–256)	3.6 (2.6–4.9)	1 (1–1)	59(29–98) (29–98)	57 (24–956)	231 (64.3)	128 (35.7)	337 (93.9)	21 (5.8)	1 (0.3)	65 (18.1)	189 (52.6)	105 (29.2)
	*p*-value	^‡^0.503	^‡^.065	^‡^.023*	^‡^0.005*	^‡^.934	^‡^.054	^‡^0.412	^‡^.001*	0.488	^§^.029*		^¶^1,000			^§^.053		

^†^Student t test, ^‡^Mann Whitney U test, ^§^Pearson Chi-square test, ^¶^Fisher Freeman Halton test, *p<0.05.

PSA: Prostate specific antigen, ASA: American Society of Anesthesiologists, PVP: Photovaporization of the Prostate, IQR: Interquartile range, SD: Standard deviation.

Cox regression analysis (“proportional hazards regression analysis”) is used as a survival model in this study to assess the relationship between multiple predictor variables and a time-to-event outcome. Univariables with p<0.2 were considered for inclusion in a multivariable Cox regression model (Table 3).

**Table 3.** Univariate and multivariate Cox-regression analysis of the factors related to reoperations.

			Age	Prostate volume (mL)	Pre-operative PSA (ng/mL)	Lasing time (minute)	Applied energy (Joule)	Applied Lasing Density (J/mL)	Surgeon experience (>100 cases)	GreenLight^TM^ PVP Device		
										80W	120W	180W
All Reoperations	Univariable	ODDS (%95 CI)	1.002 (0.968–1.036)	0.996 (0.985–1.007)	0.987 (0.905–1.077)	0.980 (0.958–1.003)	0.997 (0.994–1.000)	0.906 (0.763–1.175)	1.622 (0.891–2.953)	Reference	1.120 (0.537–2.338)	1.545 (0.599–3.526)
		*p*-value	0.920	0.437	0.777	0.093	0.084	0.258	0.114	0.682	0.763	0.408
	Multivariate	ODDS (%95 CI)	-	-	-	1.011 (0.980–1.043)	0.995 (0.990–1.000)	-	2.868 (1.139–7.219)	-	-	-
		*p*-value	-	-	-	0.474	0.045*	-	-	-	-	-
Bladder Neck Contracture	Univariable	ODDS (%95 CI)	1.029 (0.977–1.083)	0.998 (0.981–1.015)	0.966 (0.830–1.124)	0.971 (0.931–1.012	0.996 (0.991–1.002)	0.860 (0.635–1.164)	2.897 (1.155–7.263)	Reference	1.758 (0.558–5.540)	2.485 (0.682–9.058)
		*p*-value	0.283	0.813	0.652	0.165	0.208	0.328	0.023*	0.384	0.336	0.168
	Multivariate	ODDS (%95 CI)	-	-	-	0.937 (0.892–0.983)	-	-	4.031 (0.902–18.004)	Reference	0.448 (0.118–1.698)	0.725 (0.159–3.305)
		*p*-value	-	-	-	0.008*	-	-	0.068	0.370	0.238	0.678
Urethral Stricture	Univariable	ODDS (%95 CI)	1.029 (0.977–1.083)	0.813 (0.981–1.015)	0.966 (0.830–1.124)	0.971 (0.931–1.012)	0.996 (0.991–1.002)	0.860 (0.635–1.164)	1.311 (0.528–3.255)	Reference	0.879 (0.276–2.803)	1.454 (0.418–5.054)
		*p*-value	0.283	0.813	0.652	0.165	0.208	0.328	0.559	0.603	0.827	0.556
	Multivariate	ODDS (%95 CI)	-	-	-	-	-	-	-	-	-	-
		*p*-value	-	-	-	-	-	-	-	-	-	-
Persistent/ Recurrent Adenoma	Univariable	ODDS (%95 CI)	0.993 (0.936–1.053)	1.010 (0.997–1.022)	1.087 (0.993–1.190)	1.020 (0.998–1.042)	1.001 (0.996–1.007)	0797 (0.574–1.106)	0.554 (0.211–1.460)	Reference	0.571 (0.203–1.606)	0.481 (0.092–2.509)
		*p*-value	0.812	0.131	0.070	0.072	0.572	0.175	0.232	0.501	0.288	0.385
	Multivariate	ODDS (%95 CI)	-	0.996 (0.974–1.017)	1.069 (0.947–1.207)	1.023 (1.003–1.044)	-	0.732 (0.493–1.086)	-	-	-	-
		*p*-value	-	0.693	0.283	0.024*	-	0.121	-	-	-	-

p < 0.05.

PSA: Prostate specific antigen, ASA: American Society of Anesthesiologists, PVP: Photo vaporization of the Prostate, CI: Confidence interval.

## Results

During the interval of our retrospective study, 441 patients underwent PVP at our clinic. Of those, 43 patients were excluded from the analysis because of missing data or loss of follow-up. Table 1 shows the baseline characteristics of the 377 patients analyzed with regard to the inclusion criteria in this study. The median follow-up period was 61 months. The overall reoperation rate was 13.8% (52/377). The redo procedures were due to BNC, US, and persistent/recurrent adenoma in 7.7%, 5.6%, and 4.8% of cases, respectively.

The median interval until any reoperation was 36 (IQR 40–120) months. The median time to reoperation for US was 9 (4–36) months.

In reoperated patients, the laser energy delivered during PVP remained significantly lower (p<0.05). There were no significant differences in the reoperation rates between different models of PVP devices (p>0.05) (Table 2).

Lasing time, applied energy, and surgeon-experience variables were significantly associated with the overall risk of reoperations. In Cox regression multivariate analysis, the performance of surgery by an experienced surgeon was identified as a significant predictor of redo procedures (2.868 (95% CI:1.139–7.219)), while lower laser energy delivered in PVP was found weakly related to a higher total risk of reoperation (0.995 (95% CI:0.990–1.000)) (Table 3).

### Bladder neck contracture

In patients who were reoperated for BNC, lasing time and applied energy levels of PVP were significantly lower (p<0.01). This subgroup also had a markedly lower median prostate volume; however, the difference remained statistically insignificant (p=0.092). The median time to reoperation for BNC was 19 (7–76) months (Table 2). In univariable analysis, lasing time, surgeon experience, and 180W-XPS device use were found to have a significant correlation with BNC. Nevertheless, in the multivariate Cox regression model, lasing time was the only significant variable identified and inversely associated with the risk of BNC (0.937 (95% CI:0.892–0.983; p<0.01)) (Table 3).

### Urethral stricture

None of the preoperative or intraoperative variables showed statistical relevance to the occurrence of US in univariable analyses, and thus multivariate analysis was omitted.

### Persistent/Recurrent adenoma

In patients who underwent reoperations due to persistent/recurrent adenoma, the preoperative PSA level and lasing time in PVP were significantly increased (p<0.05). In these patients, we also observed that the median prostate volume was markedly high, but the difference remained non-significant (p=0.065).

In outcome analysis, a significantly higher rate of reoperation owing to adenoma was observed in patients who were operated on by less-experienced surgeons (p<0.05). The use of various PVP device models did not correlate with the risk of reoperations due to persistent/recurrent adenoma (p>0.05) (Table 2).

In the multivariate Cox regression analysis, longer lasing time was identified as a predictor of an increased risk of reoperation for persistent/recurrent adenoma in patients undergoing PVP (1.023 (95% CI: 1.003–1.044; p<0.05)). (Table 3)

## Discussion

Following an experience of using three consecutive models of GreenLight laser devices, we were interested in investigating the root causes of redo procedures in the long-term after PVP treatments in our center. After a median follow-up of 5 years, we identified an overall reoperation rate of 13.8%, which comprised procedures due to BNC, US, and persistent/recurrent adenoma in 7.7%, 5.6%, and 4.8% of cases, respectively. We did not detect noteworthy evidence for the impact of various PVP devices on the total retreatment risk. Nevertheless, we observed a higher rate of surgical reinterventions with 80W and 120W lasers (15.3% and 16.4%) in comparison to 180W (11.2%). Currently, there is no existing study on a direct comparison of the three GreenLight laser devices. The initial system (80W) was related to a higher retreatment rate as compared to TURP, ranging between 9% and 25%. Among the few studies involving high-power devices, the reoperation rates ranged between 4.3% and 15%, though long-term results are lacking [5–9].

We noted that the shortest median time-to reoperation (9 months) was due to US. Redo procedures because of BNC were observed in a wider period with a median of 25 months. Importantly, the reoperations for recurrent/persistent adenoma were recorded after a longer interval (median of 57 months), contrary to the prior studies reporting that most of the reinterventions occur during the first year after PVP [1,9].

A striking observation emerging from our data is the interesting correlation between lower laser energy usage and long-term retreatment rates. However, the present findings should be interpreted with caution, since lower energy and shorter lasing time may account for different risk factors relating to retreatments in general. While it indicates inadequate adenoma ablation in patients with large prostates, it may be linked to postoperative scar-formation due to the thermal damage on bladder neck tissue in patients with smaller adenomas.

In patients who underwent reoperation for BNC, we observed that the lasing time and applied energy levels of PVP were significantly lower, suggesting a brief surgical procedure for a smaller adenoma. This was further verified by the markedly smaller (41 mL) median prostate volume, albeit without statistical significance, among patients who required reoperation for BNC. We determined shorter lasing time as the only predictor associated with BNC, which may be linked to smaller prostate size requiring a shorter time of PVP to achieve sufficient intra-prostatic cavity. BNC remains a major cause of redo surgical procedures following PVP in a range of 1% to 12%, with an increased incidence in patients with prostates less than 40 mL, irrespective of the type of laser used for ablation [5,10,11]. BNC is characterized by scar formation and deposition of collagen and is of concern in patients treated by PVP due to the high energy delivered to the tissue. In addition to the direct thermal impact of the GreenLight laser treatment on the prostate adenoma, the surrounding tissues incur bioactive effects, termed photobiomodulation, including inflammation, collagen synthesis, and proliferation of fibroblasts [12–14]. Interestingly, research revealed that a lower dose of laser application was more likely to enhance the expression levels of scar formation-related genes and the content of the extracellular matrix than the higher dose [15,16]. Further work is needed to fully establish the link between small adenomas and BNC occurrence in PVP.

For patients requiring reoperation for US, we did not observe any difference in the demographic and perioperative features apart from the strikingly early occurrence following PVP. Once excluding meatal strictures, the 5.6% rate of de novo bulbar/penile urethral stricture after PVP in our analysis remained in line with the literature [17]. In all cases, we used the same size (22.5F) laser cystoscope, thus, we did not have comparative information in terms of instrument diameters. Although increasing endoscope size is a risk factor for stricture in transurethral procedures, Kiba et al. showed more frequent US after PVP in comparison to bipolar enucleation of the prostate (16% vs 0%) where a smaller-size instrument was used for PVP, suggesting that urethral stricture can arise from various causes [18]. Studies have priorly suggested an association between the presence of infections, increased prostate volume, repeated urethral catheterizations, and the risk of developing sclerotic changes in the urethra following all transurethral surgeries. Nevertheless, meta-analyses comparing PVP and TURP for long-term complications found no significant difference in US and did not specify any risk factors [19,20].

In patients reoperated for persistent/recurrent adenoma, our analysis revealed higher levels of PSA and longer lasing time. These differences are seemingly due to larger adenoma volumes in reoperated patients (64 mL versus 50 mL, p=0.065). Besides, the need for a greater amount of tissue ablation for efficient prostatic tissue cavitation, bleeding may frequently hamper the vaporization procedure in patients with large prostates and thus cause longer lasing time. We observed that the applied power rate was lower (2.8 J/mL versus 3.6 J/mL, p=0.054) in this subgroup, implying that lower competency of the photovaporization technique in inexperienced hands may increase the risk of redo procedures in patients with large prostates.

Surgeon experience emerged as a significant parameter for retreatment rate. The current study suggests that surgeon experience may have an impact on the occurrence of late complications. While a 20-case volume may be sufficient to acquire the vaporization skill, it takes over 100 procedures to reach an expert level of competence as defined by procedure duration and the effectiveness of volume reduction [21,22]. We assessed our data for both 75 cases and 100 cases as a cut-off in defining the experience level, and we found out that the outcomes did not change. Surprisingly, we observed that the risk of total retreatments was higher in patients who were operated on by an experienced surgeon. This paradoxical result may be related to the unselective recruitment of patients for PVP. Since the introduction of Greenlight PVP in our department, based on the clinical data supporting its efficiency, we have utilized this technique as a “true” alternative to TURP, irrespective of prostate volume. We believe that utilizing PVP as an “unconditional” alternative to TUR-P and disregarding any patient-selection criteria specific to the surgical technique might have resulted in a relatively high rate of reoperations even for surgeons with a high level of experience. Furthermore, our analysis demonstrated that patients operated by less-experienced surgeons may expect a higher risk of reoperation for larger adenomas. Studies have previously shown that novice surgeons perform less efficiently in terms of energy delivered per volume of the prostate, vaporization time/operative time ratio, and postoperative complications [9,23,24].

The current outcome data of a single center suggest that redo surgeries in the long-term are mainly due to persistent/recurrent adenoma. In this subgroup, we observed a lower (11.1%) risk of retreatment with the use of a 180W GreenLight laser device as compared to 80W and 120W devices (38.9% and 50.0%, respectively). This finding may reflect a better performance of the higher-intensity laser in terms of tissue removal. Nevertheless, we acknowledge a possible bias in the duration of follow-up among the three groups. Patients treated with the first two generations of PVP lasers are likely to have been followed up longer, and thus have an increased risk of regrowth of the adenoma. Furthermore, a lower rate of retreatment may also be linked to a cumulative experience of operative technique that ensues utilization of three generations of GreenLight PVP devices in our center.

### Limitations

The major shortcomings of the current study are related to its retrospective non-randomized design and selection bias. Use of either transabdominal or transrectal ultrasound in the estimation of pre-operative prostate size, heterogeneity between surgeons’ techniques, and varying schemes of follow-up are inherent biases, but we believe their influences remain limited and may as well reflect daily clinical practice. Likewise, this observational investigation is lacking any standard criteria used for the “re-operation” requirement. We performed Cox regression analysis (Table 3) to account for inherent differences in baseline characteristics between reintervention groups. In contrast to prior prospective randomized studies about PVP, which have focused on short-term effects and complications, we sought a comprehensive assessment of contributing factors relating to three distinct redo procedures after PVP in an unselected cohort of patients with a long-term follow-up in the same center. Since our exclusive purpose was to investigate the surgical retreatments required after PVP procedures, we intentionally did not include any objective urodynamic parameters, medical treatments, and non-surgical interventions in the pre- and post-operative data.

In conclusion, we found an overall reoperation rate of 13.8% in patients undergoing PVP with any of the three GreenLight devices. The redo procedures were due to BNC in 7.7%, US in 5.6%, and persistent/recurrent adenoma in 4.8% of the cases. We did not demonstrate any association between a PVP device model and the risk of reoperations. Multivariate analysis identified an association between BNC and shorter lasing time, whereas longer lasing time predicted an increased risk of reoperation due to adenoma. We were unable to identify any risk factors associated with US, which occurred significantly early after the PVP.

## Abbreviations

TURP: Transurethral resection of the prostate
BPH: Benign prostatic hyperplasia
PVP: Photoselective vaporisation of the prostate
US: Urethral stricture
BNC: Bladder neck contracture
KTP: Potassium titanyl phosphate
HPS: High-Performance System
XPS: Xcelerated Performance System
PSA: Prostate-Specific Antigen
ASA: American Society of Anaesthesiologists
NCSS: Number Cruncher Statistical System
